# Integration and visualization of systems biology data in context of the genome

**DOI:** 10.1186/1471-2105-11-382

**Published:** 2010-07-19

**Authors:** J Christopher Bare, Tie Koide, David J Reiss, Dan Tenenbaum, Nitin S Baliga

**Affiliations:** 1 Institute for Systems Biology, 1441 N 34th Street, Seattle, WA 98103, USA; 2 Universidade de São Paulo, Ribeirão Preto, Avenida dos Bandeirantes, 3900, Ribeirão Preto, SP, Brazil

## Abstract

**Background:**

High-density tiling arrays and new sequencing technologies are generating rapidly increasing volumes of transcriptome and protein-DNA interaction data. Visualization and exploration of this data is critical to understanding the regulatory logic encoded in the genome by which the cell dynamically affects its physiology and interacts with its environment.

**Results:**

The Gaggle Genome Browser is a cross-platform desktop program for interactively visualizing high-throughput data in the context of the genome. Important features include dynamic panning and zooming, keyword search and open interoperability through the Gaggle framework. Users may bookmark locations on the genome with descriptive annotations and share these bookmarks with other users. The program handles large sets of user-generated data using an in-process database and leverages the facilities of SQL and the R environment for importing and manipulating data.

A key aspect of the Gaggle Genome Browser is interoperability. By connecting to the Gaggle framework, the genome browser joins a suite of interconnected bioinformatics tools for analysis and visualization with connectivity to major public repositories of sequences, interactions and pathways. To this flexible environment for exploring and combining data, the Gaggle Genome Browser adds the ability to visualize diverse types of data in relation to its coordinates on the genome.

**Conclusions:**

Genomic coordinates function as a common key by which disparate biological data types can be related to one another. In the Gaggle Genome Browser, heterogeneous data are joined by their location on the genome to create information-rich visualizations yielding insight into genome organization, transcription and its regulation and, ultimately, a better understanding of the mechanisms that enable the cell to dynamically respond to its environment.

## Background

The genome encodes the physiological functions and regulatory logic by which a cell interacts with its environment. Therefore, visualization and exploration of genome-wide data in the context of their organization across the genome is critical to fully understand how an organism dynamically utilizes the information encoded in its genome to affect its physiology [1]. Recent advances in whole genome tiling arrays and next-generation sequencing technologies are providing new ways to collect genome-wide data at much higher resolution than previously possible. The ability to dynamically explore and visualize these data in a flexible, interactive and informative manner will be key to understanding these data and directly linking the mechanistic information they provide with cellular physiology.

To this end, we have created the Gaggle Genome Browser (GGB), an interactive graphical tool which enables plotting of multiple tracks of data of diverse types along the genome at multiple scales with dynamic panning and zooming. Applications initially targeted are visualization of expression and protein-DNA interaction from several measurement technologies including gene expression arrays, whole-genome tiling arrays, mass spectrometry, chromatin immunoprecipitation (ChIP-chip) and sequencing (RNA-seq or ChIP-seq) for microbial genomes.

Several genome browsers have existed for some time and our intention was not to duplicate previous efforts. With development of GGB, we focus on interactive exploration, easy access to user data, and interoperability, along with the ability to handle large sets of user-generated data gracefully. Interoperability with the Gaggle [2] framework is a central feature. By connecting to the Gaggle framework, the genome browser joins a suite of bioinformatics tools giving the researcher the power to analyze complex biological systems across several data types, from high-resolution gene expression to protein interactions, metabolic pathways, and much more.

## Implementation

**Figure 1:**
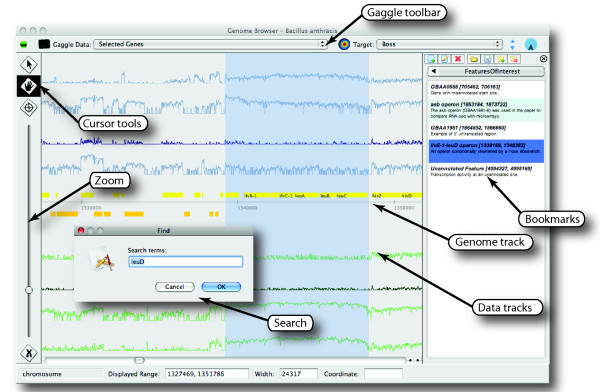
**Features of Gaggle Genome Browser**. Features of GGB include interactively panning and zooming through large amounts of user-generated data, dynamically scaling track data for effective display in limited screen resolution, integration with the Gaggle framework, search for named features, and facilities for creating and editing annotated bookmarks of regions of interest. Data shown here is RNA-seq measurements of the transcriptome of *Bacillus anthracis *by Passalacqua et al.

The Gaggle Genome Browser is written in the Java [3] programming language using the Swing UI framework and the SQLite [4] database engine. Message passing between applications is provided by the Gaggle framework. These components support the design goals of creating a cross-platform interactive graphical application that can handle large user-generated datasets, interoperate with existing tools and flexibly accommodate extension. Several key features are illustrated in Figure 1.

### Data model

**Figure 2:**
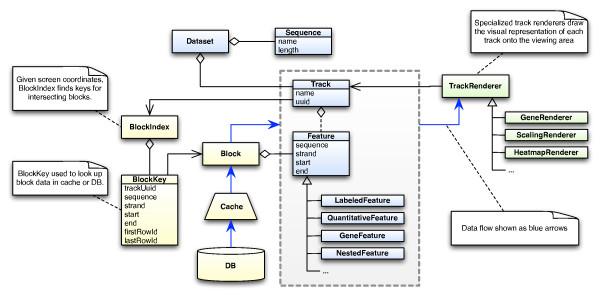
**Object model and data flow**. The basic classes of the domain model, highlighted in blue, are common to several genomics applications. A genome browser dataset consists of a list of sequences which define the coordinate system and tracks holding feature data to be plotted against those sequences. Data access (in yellow) is handled by loading contiguous blocks of feature data from an in-process database. An index can quickly determine which blocks intersect with the viewing area. Data flow (blue arrows) proceeds from the database through an LRU (least-recently-used) cache and is presented to TrackRenderers (in green) as tracks and features.

The program shares its basic data model with several genomics software packages. Its core abstractions (Figure 2, blue shading) are sequences, tracks and features, with a dataset composed of a collection of sequences and a collection of tracks. Sequences, which may be chromosomes, plasmids, contigs or any other sequences of interest, define the coordinate system on which track data is plotted. Tracks group together features from a common source. A feature is a tuple containing coordinates on the genome (sequence, strand, start, and end) augmented by additional data specific to a particular type of feature. Genes, microarray probe values, peptide measurements, or protein-DNA binding sites are all potentially features. In addition, datasets, sequences and tracks have attributes (key/value pairs) which are used to assign visual properties, provenance or other information to these entities. This flexible and extensible data model means that our software is not tied to any particular format, data type, or array platform. Any feature encoding data that can be related to position on the genome can be displayed in the browser.

### Rendering

Features are drawn on the plot by an implementation of *TrackRenderer*, an abstract class which is a key extension point of the program (Figure 2, green shading). Visualizations are implemented by extending this class. Several renderers are built into the program including those for genes, quantitative data series, and heatmaps. Renderers visually encode properties of individual features using color, shape, and other cues. Tracks are mapped to the user's choice of renderer by the track's attributes, which also hold other parameters used to configure the renderer. The open-ended data model for features with arbitrary key/value pairs dovetails with customizable renderers to support extension of the software with new visual representations or visualize unforeseen data types while confining code changes to a limited scope.

### Data access

An in-process database, SQLite, provides data storage and a command shell with a standard data manipulation language (SQL) without the need for a separate database server or the overhead of socket communication. Features are stored in a database in a separate table per track to avoid restricting the types of features the program can handle. All feature tables have columns holding coordinates on the genome. Additional columns may be included to represent properties such as quantitative values or statistical measures.

The main task of the program is to visualize features according to their position on the genome. Doing this quickly requires an efficient flow of data from the data store to the screen, with particular attention to minimizing disk access. Features are collected in tracks, which may have hundreds of thousands of features for tiling arrays or many millions for high-throughput sequencing. For large tracks, features should only be loaded into memory as needed. This is accomplished by dividing each track into contiguous blocks of features. A block may be loaded into memory and paged out as needed, providing a unit for caching and preserving a degree of locality (Figure 2, yellow shading). Like individual features, blocks are keyed by their genomic coordinates, so the program can efficiently determine which blocks intersect the visible window and schedule them to be loaded. Caching reduces the odds of rereading the same block repeatedly while keeping a tunable limit on memory usage.

### Queueing

**Figure 3:**
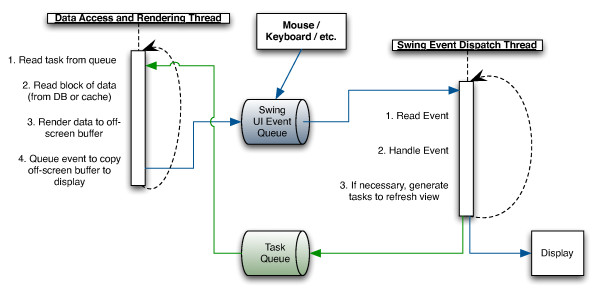
**Queueing provides responsive user interface**. The Swing event thread dispatches events from the AWT event queue (in blue) handling interaction with the user and the display. Data access and rendering tasks are placed in a task queue (in green) and executed on a separate thread. The results are rendered to an off-screen buffer which can then be rapidly copied onto the display by the UI event thread.

Both data access and rendering take place off the Swing event dispatch thread so the user interface remains responsive to user input. Data access and rendering are time-consuming and can lag behind the events generated by the UI. This mismatch of rates is handled using the queueing arrangement shown in Figure 3. To protect Swing's event dispatch thread from long-running tasks, GGB creates a separate task queue for data access and rendering tasks. A worker thread takes tasks from this second queue, rendering to an off screen image buffer. Only the copy to the display need be performed by the Swing event dispatch thread.

### Gaggle connectivity

The genome browser is a stand-alone desktop program, but its power is multiplied when used within the Gaggle framework. The Gaggle framework [2] provides data exchange between bioinformatics applications using a handful of universal data types which cover a wide range of use cases within the systems biology domain. Gaggle data types include *lists *of identifiers, *tuples *(sets of key/value pairs), *matrices *containing numeric data, and *networks*.

Applications become part of the Gaggle framework by implementing the ability to send and receive messages containing these data types. Software tools connected through the Gaggle include: Cytoscape [5], a network viewer; MeV [6], an application for analysis, visualization and data-mining of large-scale genomic data; the R Project [7] for statistical computing; and Bioinformatics Resource Manager [8], a data management, analysis and integration environment for systems biology. Firegoose [9], a toolbar for the Firefox browser, further extends the Gaggle environment to web resources such as: KEGG [10], for metabolic pathways; STRING [11], for protein interactions; and DAVID [12], for functional annotations.

The effect of Gaggle connectivity is that the genome browser can focus on visualization without taking on the impossible task of reimplementing all the functionality of the various Gaggle-connected resources.

### Architecture

The application is factored into modular components which communicate with each other through events. The application exposes an API (currently in prototype form) for use by components, plug-ins and scripting. This API is used to implement Gaggle integration and forms the basis for an R package that enables control of the genome browser by commands within the R environment.

Heer et al. [13] present a system of software design patterns for visualization, several of which are applied in GGB. The general structure of the application loosely follows the Reference Model design pattern, a specialization of the Model-View-Controller pattern that further divides the model into the underlying data model and a visualization, a mapping onto visual properties such as color, shape, and position.

When a single track has features numbering in the tens or hundreds of millions, allocating an object for each feature is prohibitively inefficient. For this reason, features in GGB are typically Flyweights [14]. For all features of a track, a single flyweight feature provides an object oriented interface backed by parallel arrays. An individual feature then reduces to an index into the arrays and iterating through features simply amounts to incrementing the index. Thus memory is used efficiently and features nearby in the genome are also nearby in memory boosting cache locality.

Software designed to meet the fluid requirements of research applications must be flexible, adapting to changing needs and a range of usage styles [15]. The goal of our architecture is to provide the necessary flexibility through extensibility and interoperability with a range of tools from point-and-click web resources to sophisticated environments such as R.

## Results

**Figure 4:**
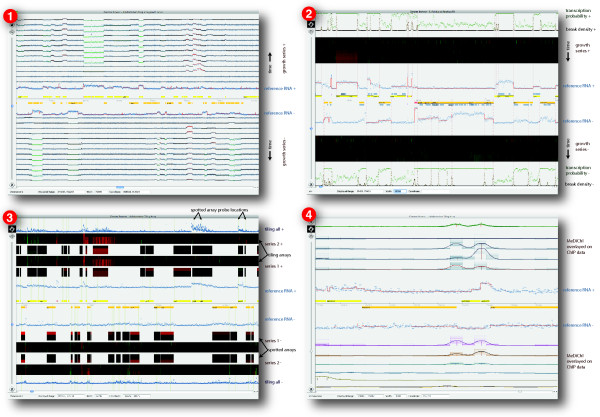
**Gallery of Gaggle Genome Browser visualizations**. (1) H. salinarum growth series showing 14 tracks of strand sensitive tiling array data taken as a time series during growth. The track nearest the horizontal axis shows reference RNA, while the remainder of the tracks are log ratios relative to the reference. Segmentation, overlaid on the reference RNA in red, computationally delimits transcriptional units. Ratios are also overlaid with segmentation, using red to indicate increased expression and green for decreased expression relative to the reference for that segment. This view shows about 200 thousand features out of 7.25 million in the whole dataset. (2) A view supporting curated annotation of transcriptional start and termination sites. Heatmaps are used to represent tiling array data relative to the reference condition, shown with blue circles overlaid with segmentation in red. Computed boundaries of transcription are drawn as dashed verticals with supporting statistics shown in brown and green along the outer edge. Blue blocks show PFAM domains. Dark blue bars show computationally predicted operons. (3) A comparison of array platforms. Data from different tiling array platforms is compared to spotted expression arrays. On the outer edge, we overlay all time points from both replicates giving some idea about the distribution of values at each point. (4) *MeDiChI *profiles and predicted binding sites overlay multiple replicates of ChIP-chip data for several transcription factors, showing TF binding sites in relation to genes and transcription data.

The architecture described above results in a versatile tool for visualizing genome related data. Data can be imported from a wide variety of sources including the GFF standard file format [16] and the UCSC Genome Browser [17] through a wizard interface. Once imported, data can be visualized at multiple zoom levels and navigated by scrolling, searching, or jumping to directly to bookmarked regions of interest. Computationally oriented users can leverage the powerful data manipulation features of the R statistical environment [7] or SQL. Track data can be visualized using several renderers, including one for heatmaps and a scaling renderer that changes representation based on zoom level (Figure 4).

**Figure 5:**
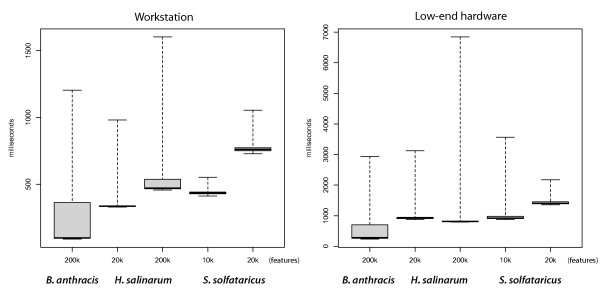
**Comparison of image rendering times for different datasets and zoom levels**. Complexity of the visual representation has a large effect on rendering time, as does the number of features visible in the viewing area. Whiskers indicate the range of rendering times, while boxes show the middle two quartiles. Rendering is usually under one second even for very complex renderings on workstation class hardware and slower but still acceptable on a low-end machine. Slower rendering times are associated with cache misses. The *B. anthracis *dataset (43 million features total, shown in figure 1) is the fastest to render, benefiting from the scaling renderer that adapts to zoom level. The *H. salinarum *dataset (7.25 million features total, shown in panel 1 of figure 4) is of moderate complexity with 30 total tracks. Shown are rendering times for a zoomed in view with 20 thousand features visible and a zoomed out view with 200 thousand features visible. The *S. solfataricus *dataset, (27 million features total, shown in panel 2 of figure 4) with 39 tracks including heatmaps shows slowest rendering times. We show rendering times for 10 thousand and 20 thousand features. While more zoomed out views of datasets with heatmaps render slowly, the program remains responsive at all times.

Fast graphical rendering is difficult to achieve in cross-platform applications. GGB delivers frame rates sufficient for interactive visualization for datasets in our experience (Figure 5). Leaving aside window dimensions, rendering speed depends mainly on the number of features visible in the viewing area (zoom level) and the complexity of their visual representation. For example, a heatmap takes longer to draw than a line plot. Rendering speed shows no discernible dependence on the size of the database or total number of features. In all cases, even when rendering slows, the program remains responsive to user input, resulting in good subjective performance. The program has been tested with datasets as large as 500 million features, equivalent to covering human chromosome 1 at single nucleotide resolution. It runs comfortably with a heap size of 256 MB resulting in a total memory footprint of about 350 MB independent of total data size. We tested the program both on a workstation class machine (2.66 GHz Quad-core, 8 GB main memory) and on modest hardware (1.92 GHz CPU and 1 GB main memory) finding adequate performance even on the low-end machine. Further performance measurements are available on the GGB website [18].

A pair of case studies demonstrate the use of the Gaggle Genome Browser in analyzing diverse, complex and large datasets to discover biologically meaningful insights. The first focuses on a discovery of internal promoters that was made possible by extensive manual exploration and curation of the transcriptome structure in conjunction with protein-DNA interactions and interactive statistical analysis in R. The second example illustrates that GGB gracefully handles 8 tracks of single nucleotide resolution next-generation sequencing data, with a total of over 45 million features in a 358 MB database.

### Case study: Discovery of a conditionally active promoter inside a coding sequence of the succinate dehydrogenase operon

Our genome browser was developed in conjunction with a study of the transcriptome structure of *Halobacterium salinarum*[19]. (GEO GSE13150) Transcription and protein-DNA binding were measured using whole-genome tiling arrays at several time points over the growth curve. This data was used to revise computationally predicted genes and discover new protein-coding regions and non-coding RNAs. A segmentation algorithm was used to find breaks in transcription defining transcription start and termination sites and operon structure. In some cases, these structures were shown to change over the growth curve revealing the dynamic nature of transcription. ChIP-chip data was used to relate changes in transcription to the binding of transcription factors.

An important discovery made in this study was that there is a higher than expected incidence of transcription initiation inside operons, including within the coding sequences of member genes. The discovery of these and other novel insights required the Genome Browser to support extensive interactive exploration of transcriptome structure changes and protein-DNA interactions in context of the genome map and to integrate well with *R*, which was used to process raw signal data and compute derived data such as segmentation and probable binding sites. Part of this analysis is reconstructed here to illustrate the features of the genome browser and its interoperability with other tools. The reader is encouraged to follow along with the detailed instructions on the Gaggle website [18].

In brief, we will focus on an observation that there is a growth-associated transcriptome structure change within the 4 gene operon for succinate dehydrogenase. We wished to investigate whether the break in transcription associated with this event coincides with the location of a transcription factor binding site (TFBS) for the transcription factor TFBd. We assayed DNA binding for TFBd using chromatin immunoprecipitation followed by two different whole genome tiling array platforms, an in-house array with 500 base-pair resolution (GEO GSE7045) [20] and a higher resolution Nimblegen tiling array (GEO GPL8468). Part of the intent was to test the sufficiency of the 500 bp array to predict binding sites.

Given the low resolution of this data it is difficult to evaluate visually whether there is a statistically significant TFBd binding event in the vicinity of the putative internal promoter. The resolution to which specific binding sites in a ChIP-chip assay can be identified is limited by: (a) the resolution of the tiling microarray, and (b) the variable sizes of the immunoprecipitated DNA fragments. Together, these issues can influence the accuracy of localizing TFBSs from ChIP-chip data. To address these technical challenges we developed *MeDiChI *[21], to precisely localize binding locations at a resolution higher than the tiling array probe spacing. *MeDiChI *produces a model fit and peaks representing locations and intensities of predicted TFBSs.

**Figure 6:**
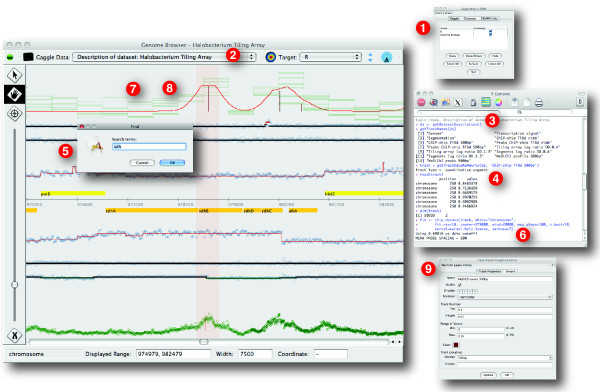
**Processing track data in R with the *MeDiChI *ChIP-chip deconvolution algorithm**. GGB can be used in conjunction with the R environment for statistical computing through the Gaggle framework. After connecting both the genome browser and R to the Gaggle framework (1) we broadcast a description of the dataset using the Gaggle toolbar (2) from the genome browser to R. We can then inspect the dataset in R (3) and load track data into the R environment (4). Locating the region of interest, the sdh operon, using the search feature (5) we then apply *MeDiChI*'s chip.deconv function to the track (6). From R, we broadcast data to the genome browser which then creates new tracks for model fit (7) and peaks (8). We then adjust the visual properties of the new tracks (9) to display predicted transcription factor binding sites.

The workflow used to derive these results started with a track of ChIP-chip data in GGB, which was transmitted to R for analysis with *MeDiChI*. Products of that analysis are then transmitted back to the Genome Browser for visualization. Data transfer was done by establishing a connection between the Genome Browser and R using the Gaggle framework (Figure 6, step 1) assisted by a library of supporting functions called *genome_browser_support.R*.

First, a description of the dataset was broadcasted from the genome browser to *R*. (Figure 6, step 2) This descriptive data about the dataset and its tracks includes information necessary to access the Genome Browser's underlying database directly from within R. Sharing the database between R and GGB amounts to a form of pass-by-reference which avoids pushing large data structures through Gaggle's messaging protocol, efficiently passing pointers instead. The 500 bp resolution ChIP-chip track was selected by interrogating the dataset metadata (Figure 6, step 3) and the track data loaded into the R environment (Figure 6, step 4).

*MeDiChI *was then applied to the ChIP-chip data over the genome region containing the succinate dehydrogenase operon. The Genome Browser's search function was used to find the coordinates of this region (Figure 6, step 5) for input into *MeDiChI*'s *chip.deconv *function (Figure 6, step 6). The two kinds of derived data from this analysis -- a profile which represents the best fit of *MeDiChI*'s model to the data and a list of peaks at the predicted sites of protein-DNA binding -- were broadcasted back to the Genome Browser for visualization (Figure 6, steps 7 and 8). The genome browser's *Track Visual Properties Editor *was then used to set rendering options for the new tracks (Figure 6, step 9).

By visualizing the raw and processed transcriptome and ChIP-chip data we observed a binding site for TFBd close to the 3' end of *sdhB*. As growth progresses, a break in the transcript for this operon appears, suggesting that there are condition-dependent alternative transcripts for this operon. Based on such interactive analysis we hypothesized the presence of transcriptional promoters in at least 40% of all operons in *H. salinarum NRC-1*. Several of these were validated with promoter-GFP fusion assays. In sum, the interactive exploration of the diverse genomic datasets within GGB was crucial in formulating hypotheses that led to the discovery of extraordinary complexity in prokaryotic transcription.

### Case study: Inspection of transcriptome structure through interactive exploration of 350 MB of next-generation sequencing data

Passalacqua, et al. [22] mapped the transcriptome of *Bacillus anthracis *at single-nucleotide resolution using high-throughput sequencing technology (RNA-seq). To test our ability to handle data at this density, we imported the eight tracks of single nucleotide coverage data for this 5.4 megabase genome. As represented in the genome browser, these ~43 million features occupy 358 MB. GGB renders this data responsively and maintains a moderate memory footprint (~350 MB when run with a maximum heap size of 256 MB). This visualization is shown in Figure 1 and is available as a live demo on the Gaggle website [18]. Bookmarks annotating a region of the genome with a name and short description are included to allow quick navigation to features discussed in the paper.

Because data come from instruments, public repositories and other software in a bewildering variety of formats, GGB allows users to import data using their choice of tools and scripting languages. In this example, a short Python script created the SQLite data file taking as input sequence annotation data from NCBI (refseq IDs NC_007530 and NC_007322) and coverage data provided by the authors in tab-delimited text files. The same result could have been achieved using R and Gaggle or SQLite's command line shell as well as importing from formatted text files.

## Discussion

### Related work

The insight to be gained by visualizing biological data plotted along the scaffold of the genome has long been recognized. There are several established genome browsers. Recent developments in both laboratory techniques and computing technologies have motivated several new entries in this category as well.

The UCSC Genome Browser [17] and its microbial counterpart [23] are the most widely known. A major asset of these popular resources is that each model organism is augmented with a rich collection of curated track data. The Table Browser feature [24] provides user-level and programmatic access to this data making these services excellent data sources as well as visualization tools. A wizard interface to acquire chromosome layout data from these sources is built into GGB, as is a link in the right-click menu that opens the currently selected or displayed region in the UCSC Genome Browser, for available organisms. The UCSC Genome Browsers are page-based web applications in which images are generated on the server, which places some limits on interactivity. For example, pan and zoom operations require a page refresh.

Rich web technologies can provide a more interactive experience with all the advantages of a remotely hosted web application. X:Map [25] is an impressive example, using the Google Maps API to interactively scroll and zoom through pre-rendered image tiles. JBrowse [26] demonstrates the efficiency of AJAX based client-side rendering. Like its predecessor GBrowse [27], JBrowse is backed by the BioPerl library. Web applications benefit from handling data and CPU intensive operations on the server. However, requiring users to upload their own data to another institution's server has implications for security and bandwidth. Even so, a rich web implementation remains an appealing option especially one taking advantage of the enhanced vector graphics capabilities of Flash, SVG or HTML5.

Our choice to build the GGB as a desktop application was largely motivated by the need to support large user-generated datasets. Java was chosen to complement other Gaggle tools and for cross-platform support. Other desktop genome browsers also implemented in Java include the Apollo Genome Annotation Curation Tool [28], which was used to annotate the fly genome. Affymetrix released the Integrated Genome Browser (IGB) [29] and the supporting Genoviz SDK [30] as open-source projects. The Broad Institute provides Argo [31] and IGV (Integrative Genomics Viewer) [32]. The recently published MochiView [33] is an excellent tool which integrates support for motif detection based on ChIP data. Both IGV and MochiView emphasize handling of high-density data types.

The central distinguishing feature of GGB is Gaggle integration, which offers a wealth of options for exploring different types of data in relation to location on the genome. Also, GGB's flexibility in track rendering enables visualizations that would be difficult to reproduce using any other software. This flexibility derives mainly from a variety of customized renderers and a free-form approach to laying out tracks. Multiple data series may be drawn on top of each other or partially overlapping, using transparency and z-order to convey additional information. Finally, GGB offers a point-and-click wizard for creating a new project based on any genome curated by UCSC. A similar feature using NCBI as a data source is under development.

### Future directions

GGB remains a work in progress. Various directions for future development are under consideration, dependent on user demand. Primarily, GGB is designed to be ready for extension in the kinds of visualizations and the data types being visualized as well as additional avenues of interoperability.

A feature currently lacking is the ability to work directly with sequence data. Directly storing and looking up sequence data is one option, but we hope that integration, through Gaggle or other mechanisms, with existing applications may serve this purpose. We also do not display exons, given the emphasis in our own research on microbes. New data types and renderers supporting exon display could be implemented through existing extension points.

Interoperability is a particular emphasis in our development efforts. Building on Gaggle integration, we are prototyping a library of R functions for communicating with GGB. Further developing this library could help computational scientists use the sophisticated analysis and data manipulation capabilities of R and Bioconductor [34] together with visualization in the genome browser. For other users, enhanced point-and-click import of data from widely used sources such as the UCSC Genome Browser, NCBI Entrez [35], or DAS (Distributed Annotation System) [36] would be most important. Extending these mechanisms would allow the software to be applied to a larger variety of organisms and to more easily take advantage of the wealth of existing resources.

To address rendering performance in zoomed out views, where data points greatly outnumber the pixels available in which to display them, our scaling renderer aggregates features during rendering by computing ranges and means. Precomputing these aggregates at predefined scales could further increase performance. Buffering and reusing previously rendered image tiles would decrease CPU load while increasing opportunities for parallelism.

## Conclusions

GGB provides the researcher with an interactive visualization tool for any data type that can be related to a location on the genome. Through the Gaggle framework, GGB can function as part of a powerful suite of bioinformatics tools able to exchange data with analysis software, other visualizations, and several public data sources.

Biological data can be joined together along several axes. Expression, interactions, and functions can be merged by gene or protein identifiers. Sequence similarity can form the basis of mapping data across organisms by orthology. In the Gaggle Genome Browser, heterogeneous data is joined by its location on the genome to create information-rich visualizations yielding insight into transcription and its regulation and, ultimately, a better understanding of the mechanisms that enable the cell to dynamically respond to its environment.

## Availability and requirements

The Gaggle Genome Browser is written in Java (1.6 or higher) and depends on the SQLite database engine. It runs on Linux, Mac OS X, and Windows, either as a stand-alone application or by web-start. We recommend a minimum screen resolution of 1024 × 768 and at least 1 GB of memory.

The program and its source code are released under the terms of the LGPL http://www.gnu.org/copyleft/lesser.html and are available on the Gaggle web site [18]. Demos, documentation and forms for submitting bug reports and feature requests are also linked from this page.

## Authors' contributions

**JCB **designed and implemented the software and drafted the manuscript. **TK **performed the characterization of the *H. salinarum *transcriptome that led to the case study. **DJR **performed the analysis of the *H. salinarum *transcriptome that led to the case study and assisted in R and *MeDiChI *integration. **DT **reviewed software design. **NSB **conceived and initiated the project, provided direction and feedback on the quality of results and software design and crafted the case study and drafted the manuscript. All authors reviewed and approved the manuscript and reviewed early versions of the software.
